# Supplementary material for: Medication Adherence Measurement in Chronic Diseases: A State-of-the-Art Review of the Literature
Source: Nurs Rep. 2025 Oct 16;15(10):370. doi: 10.3390/nursrep15100370 (PMC12567100; doi:10.3390/nursrep15100370)
Supplement: Supplementary file 1 [file nursrep-15-00370-s001.zip › nursrep-3781226-supplementary.pdf]

Supplementary File S1. Search Strategy

The literature search was developed in consultation with an experienced medical librarian and adapted for each database. Searches were performed in PubMed, EMBASE (via Ovid), and Web of Science Core Collection on August 5, 2024, covering publications from August 1, 2019 to July 30, 2024.

The strategy combined controlled vocabulary terms (e.g., MeSH, Emtree) and free-text keywords for *medication adherence* and *chronic disease*. The search was limited to English-language publications and human subjects. No study design filters were applied to maximize retrieval.

Example: PubMed Search Strategy

("Medication Adherence"[Mesh] OR "medication adherence"[tiab] OR "medication compliance"[tiab]

OR "treatment adherence"[tiab] OR "drug adherence"[tiab] OR "drug compliance"[tiab])

AND

("Chronic Disease"[Mesh] OR "chronic disease*"[tiab] OR "long term condition*"[tiab]

OR "long-term illness*"[tiab] OR "non-communicable disease*"[tiab]

OR "cardiovascular disease"[tiab] OR diabetes[tiab] OR hypertension[tiab] OR cancer[tiab]

OR asthma[tiab] OR COPD[tiab] OR "chronic kidney disease"[tiab])

Filters: Publication date from 2019/08/01 to 2024/07/30; Humans; English.

Example: EMBASE (Ovid) Search Strategy

('medication adherence'/exp OR 'medication adherence':ti,ab OR 'medication compliance':ti,ab

OR 'treatment adherence':ti,ab OR 'drug adherence':ti,ab OR 'drug compliance':ti,ab)

AND

('chronic disease'/exp OR 'chronic disease*':ti,ab OR 'long term condition*':ti,ab

OR 'non communicable disease*':ti,ab OR 'cardiovascular disease':ti,ab

OR diabetes:ti,ab OR hypertension:ti,ab OR cancer:ti,ab OR asthma:ti,ab OR copd:ti,ab

OR 'chronic kidney disease':ti,ab)

AND [english]/lim AND [humans]/lim

AND [2019-08-01]/sd NOT [2024-07-31]/sd

Example: Web of Science Core Collection Search Strategy

TS=("medication adherence" OR "medication compliance" OR "treatment adherence" OR "drug adherence" OR "drug compliance")

AND

TS=("chronic disease*" OR "long term condition*" OR "non communicable disease*" OR "cardiovascular disease" OR diabetes OR hypertension OR cancer OR asthma OR COPD OR "chronic kidney disease")

Refined by: LANGUAGES: (English) AND DOCUMENT TYPES: (Article)

Timespan: 2019-08-01 to 2024-07-30.

Indexes: SCI-EXPANDED, SSCI, ESCI.

Note:

The search syntax was adapted for the indexing and field codes of each database. Reference lists of included studies were also screened to identify any additional eligible publications.
